# Association of Altered Liver Enzymes With Alzheimer Disease Diagnosis, Cognition, Neuroimaging Measures, and Cerebrospinal Fluid Biomarkers

**DOI:** 10.1001/jamanetworkopen.2019.7978

**Published:** 2019-07-31

**Authors:** Kwangsik Nho, Alexandra Kueider-Paisley, Shahzad Ahmad, Siamak MahmoudianDehkordi, Matthias Arnold, Shannon L. Risacher, Gregory Louie, Colette Blach, Rebecca Baillie, Xianlin Han, Gabi Kastenmüller, John Q. Trojanowski, Leslie M. Shaw, Michael W. Weiner, P. Murali Doraiswamy, Cornelia van Duijn, Andrew J. Saykin, Rima Kaddurah-Daouk

**Affiliations:** 1 Center for Computational Biology and Bioinformatics, Indiana Alzheimer Disease Center, Department of Radiology and Imaging Sciences, Indiana University School of Medicine, Indianapolis; 2 Department of Psychiatry and Behavioral Sciences, Duke University, Durham, North Carolina; 3 Department of Epidemiology, Erasmus Medical Centre, Rotterdam, the Netherlands; 4 Institute of Bioinformatics and Systems Biology, Helmholtz Zentrum München, German Research Center for Environmental Health, Neuherberg, Germany; 5 Duke Molecular Physiology Institute, Duke University, Durham, North Carolina; 6 Rosa & Co LLC, San Carlos, California; 7 University of Texas Health Science Center at San Antonio, San Antonio; 8 German Center for Diabetes Research, Neuherberg, Germany; 9 Department of Pathology and Laboratory Medicine, University of Pennsylvania, Philadelphia; 10 Center for Imaging of Neurodegenerative Diseases, Department of Radiology, San Francisco Veterans Affairs Medical Center and University of California, San Francisco; 11 Duke Institute of Brain Sciences, Duke University, Durham, North Carolina; 12 Department of Medicine, Duke University, Durham, North Carolina; 13 Nuffield Department of Population Health, Oxford University, Oxford, United Kingdom

## Abstract

**Importance:**

Increasing evidence suggests an important role of liver function in the pathophysiology of Alzheimer disease (AD). The liver is a major metabolic hub; therefore, investigating the association of liver function with AD, cognition, neuroimaging, and CSF biomarkers would improve the understanding of the role of metabolic dysfunction in AD.

**Objective:**

To examine whether liver function markers are associated with cognitive dysfunction and the “A/T/N” (amyloid, tau, and neurodegeneration) biomarkers for AD.

**Design, Setting, and Participants:**

In this cohort study, serum-based liver function markers were measured from September 1, 2005, to August 31, 2013, in 1581 AD Neuroimaging Initiative participants along with cognitive measures, cerebrospinal fluid (CSF) biomarkers, brain atrophy, brain glucose metabolism, and amyloid-β accumulation. Associations of liver function markers with AD-associated clinical and A/T/N biomarkers were assessed using generalized linear models adjusted for confounding variables and multiple comparisons. Statistical analysis was performed from November 1, 2017, to February 28, 2019.

**Exposures:**

Five serum-based liver function markers (total bilirubin, albumin, alkaline phosphatase, alanine aminotransferase, and aspartate aminotransferase) from AD Neuroimaging Initiative participants were used as exposure variables.

**Main Outcomes and Measures:**

Primary outcomes included diagnosis of AD, composite scores for executive functioning and memory, CSF biomarkers, atrophy measured by magnetic resonance imaging, brain glucose metabolism measured by fludeoxyglucose F 18 (^18^F) positron emission tomography, and amyloid-β accumulation measured by [^18^F]florbetapir positron emission tomography.

**Results:**

Participants in the AD Neuroimaging Initiative (n = 1581; 697 women and 884 men; mean [SD] age, 73.4 [7.2] years) included 407 cognitively normal older adults, 20 with significant memory concern, 298 with early mild cognitive impairment, 544 with late mild cognitive impairment, and 312 with AD. An elevated aspartate aminotransferase (AST) to alanine aminotransferase (ALT) ratio and lower levels of ALT were associated with AD diagnosis (AST to ALT ratio: odds ratio, 7.932 [95% CI, 1.673-37.617]; *P* = .03; ALT: odds ratio, 0.133 [95% CI, 0.042-0.422]; *P* = .004) and poor cognitive performance (AST to ALT ratio: β [SE], −0.465 [0.180]; *P* = .02 for memory composite score; β [SE], −0.679 [0.215]; *P* = .006 for executive function composite score; ALT: β [SE], 0.397 [0.128]; *P* = .006 for memory composite score; β [SE], 0.637 [0.152]; *P* < .001 for executive function composite score). Increased AST to ALT ratio values were associated with lower CSF amyloid-β 1-42 levels (β [SE], −0.170 [0.061]; *P* = .04) and increased amyloid-β deposition (amyloid biomarkers), higher CSF phosphorylated tau_181_ (β [SE], 0.175 [0.055]; *P* = .02) (tau biomarkers) and higher CSF total tau levels (β [SE], 0.160 [0.049]; *P* = .02) and reduced brain glucose metabolism (β [SE], −0.123 [0.042]; *P* = .03) (neurodegeneration biomarkers). Lower levels of ALT were associated with increased amyloid-β deposition (amyloid biomarkers), and reduced brain glucose metabolism (β [SE], 0.096 [0.030]; *P* = .02) and greater atrophy (neurodegeneration biomarkers).

**Conclusions and Relevance:**

Consistent associations of serum-based liver function markers with cognitive performance and A/T/N biomarkers for AD highlight the involvement of metabolic disturbances in the pathophysiology of AD. Further studies are needed to determine if these associations represent a causative or secondary role. Liver enzyme involvement in AD opens avenues for novel diagnostics and therapeutics.

## Key Points

**Question:**

Are liver function markers associated with cognition and the “A/T/N” (amyloid, tau, and neurodegeneration) biomarkers for Alzheimer disease?

**Findings:**

In this cohort study of 1581 older adults, elevated aspartate aminotransferase to alanine aminotransferase ratios were associated with diagnosis of Alzheimer disease, poor cognition, lower cerebrospinal fluid levels of amyloid-β 1-42, increased amyloid-β deposition, higher cerebrospinal fluid levels of phosphorylated tau and total tau, and reduced brain glucose metabolism. Lower levels of alanine aminotransferase were associated with increased amyloid-β deposition, reduced brain glucose metabolism, greater brain atrophy, diagnosis of Alzheimer disease, and poor cognition.

**Meaning:**

Consistent associations of serum-based liver function markers with Alzheimer disease biomarkers highlight the involvement of metabolic disturbances in the pathophysiology of Alzheimer disease.

## Teaser

This cohort study examines whether liver function markers are associated with cognitive dysfunction and the amyloid, tau, and neurodegeneration biomarkers in individuals with Alzheimer disease.

## Introduction

Metabolic activities in the liver determine the state of the metabolic readout of peripheral circulation. Mounting evidence suggests that patients with Alzheimer disease (AD) display metabolic dysfunction.^1^ Clinical studies suggest that impaired signaling, energy metabolism, inflammation, and insulin resistance play a role in AD.^2,3^ This observation is in line with the observation that many metabolic disorders (eg, diabetes, hypertension, obesity, and dyslipidemia) are risk factors for AD.^4^ This evidence highlights the importance of the liver in the pathophysiological characteristics of AD. Focused investigation to assess the role of liver function in AD and its endophenotypes is required to bridge the gap between these observations.

Peripheral blood levels of biochemical markers including albumin, alkaline phosphatase, alanine aminotransferase (ALT), aspartate aminotransferase (AST), and total bilirubin are used to assess liver function. Alanine aminotransferase and AST are used in general clinical practice to measure liver injury^5,6^ and are factors associated with cardiovascular and metabolic diseases,^7,8^ known risk factors of AD and cognitive decline.^9,10^ Given this fact, it is conceivable that aminotransferases are surrogate biomarkers of liver metabolic functioning. A systematic search yielded few reports related to research in humans linking peripheral biomarkers of liver functioning to central biomarkers related to AD including amyloid-β and tau accumulation, brain glucose metabolism, and structural atrophy.

We investigated the association of peripheral liver function markers with AD diagnosis, cognition, and biomarkers of AD pathophysiological characteristics including neuroimaging (magnetic resonance imaging [MRI] and position emission tomography [PET]) and cerebrospinal fluid (CSF) from older adults in the AD Neuroimaging Initiative (ADNI) cohort. The AD biomarkers were selected and defined consistent with the National Institute on Aging–Alzheimer Association Research Framework (amyloid, tau, and neurodegeneration [A/T/N]) for AD biomarkers that defines 3 general groups of biomarkers based on the nature of pathologic process that each measures.^11^

## Methods

### Study Population

Individuals in this study were participants of ADNI. The initial phase (ADNI-1) was launched in 2003 to test whether serial MRI markers, PET markers, other biological markers, and clinical and neuropsychological assessment could be combined to measure the progression of mild cognitive impairment (MCI) and early AD. The initial phase was extended to subsequent phases (ADNI-GO, ADNI-2, and ADNI-3) for follow-up of existing participants and additional new enrollments. Inclusion and exclusion criteria, clinical and neuroimaging protocols, and other information are reported elsewhere.^12,13,14^ Demographic and clinical information, raw data from neuroimaging scans, CSF biomarkers, information on *APOE* status, and cognitive scores were downloaded from the ADNI data repository.^12^ Baseline data were collected from September 1, 2005, to August 31, 2013. Written informed consent was obtained at enrollment, which included permission for analysis and data sharing. This study was approved by each participating site’s institutional review board. This report followed the Strengthening the Reporting of Observational Studies in Epidemiology (STROBE) reporting guidelines for cohort studies.

### Liver Function Markers

Five laboratory tests were downloaded from the ADNI data repository and used in the study: total bilirubin, albumin, alkaline phosphatase, ALT, and AST. The liver function markers followed a normal distribution after log transformation. For each marker, participants with values greater or smaller than 4 SDs from its mean value were considered outliers and were removed. To determine if outliers had a significant effect on our findings we performed a sensitivity analysis and observed few differences (or slightly more significant), if any, in results when including outliers (eTable 1 in the Supplement).

### Dementia Diagnosis

Participants in ADNI were classified as cognitively normal controls (CN) or having significant memory concerns (SMC), MCI, or mild clinical AD. Criteria for classification were as follows: Mini-Mental State Examination score range (range, 0 [worst] to 30 [best]) for CN and MCI was 24 to 30, and for AD was 20 to 26; and overall Clinical Dementia Rating score (range for each, 0 [best] to 3 [worst]) for CN was 0, for MCI was 0.5 with a mandatory requirement of memory box score of 0.5 or greater, and for AD was 0.5 or 1.^15^ Cognitively normal controls did not have any significant impairment in cognition or activities of daily living. Participants with SMC had normal cognition and no significant impairment in activities of daily living, but had a score of 16 or more on the first 12 items of the self-report version of the Cognitive Change Index (range, 12 [no change] to 60 [severe change]).^16^ Participants with MCI had cognitive impairments in memory and/or other domains but were able to perform activities of daily living and did not qualify for a diagnosis of dementia.^15^ Participants with AD had to meet the National Institute of Neurological and Communicative Disorders and Stroke–AD and Related Disorders Association criteria for probable AD.^17^ Participants from the ADNI-1 cohort with MCI were all classified as late MCI, with a memory impairment approximately 1.5 SD below education-adjusted norms. In the ADNI-GO and ADNI-2 cohort, participants with MCI were classified as either early MCI, with a memory impairment approximately 1 SD below education-adjusted norms, or late MCI (same criteria as in ADNI-1). Both ADNI-1 and ADNI-GO and ADNI-2 participants met the criteria for amnestic MCI, but many in the ADNI-GO and ADNI-2 cohort included the earlier stage MCI designation (ie, early MCI).^18^

### Cognition

Composite scores were used to measure memory and executive functioning. A memory composite score was created from the following: memory tasks from the Alzheimer Disease Assessment Scale–cognitive subscale, the Rey Auditory Verbal Learning Test, memory components of the Mini-Mental State Examination, and the Logical Memory task.^19^ An executive function composite score included the following: Wechsler Adult Intelligence Scale–Revised Digit Symbol Substitution task and Digit Span backward task, Trail Making Test Parts A and B, category fluency (animals and vegetables), and 5 clock drawing items. Composite scores have a mean of 0 and an SD of 1.^20^

### Neuroimaging Processing

#### MRI Scans

Baseline T1-weighted brain MRI scans were acquired using a sagittal 3-dimensional magnetization prepared rapid gradient echo scans following the ADNI MRI protocol.^21,22^ As previously detailed, FreeSurfer, version 5.1, a widely used automated MRI analysis approach, was used to process MRI scans and extract whole-brain and region-of-interest (ROI)–based neuroimaging endophenotypes including volumes and cortical thickness determined by automated segmentation and parcellation.^23,24,25^ The cortical surface was reconstructed to measure thickness at each vertex. The cortical thickness was calculated by taking the Euclidean distance between the gray and white boundary and the gray and CSF boundary at each vertex on the surface.^26,27,28^

#### PET Scans

Preprocessed fludeoxyglucose (FDG) F 18 (^18^F) and [^18^F]florbetapir PET scans (coregistered, averaged, standardized image and voxel size, and uniform resolution) were downloaded from the ADNI Laboratory of Neuro Imaging (LONI) site^12^ as described in previously reported methods for acquisition and processing of PET scans.^23,29^ For [^18^F]FDG-PET, scans were intensity normalized using a pons ROI to create [^18^F]FDG standardized uptake value ratio (SUVR) images. For [^18^F]florbetapir PET, scans were intensity normalized using a whole cerebellum reference region to create SUVR images.

#### CSF Biomarkers

The ADNI generated CSF biomarkers (amyloid-β 1-42, total tau [t-tau], and phosphorylated tau_181_ [p-tau_181_]) in pristine aliquots of 2401 ADNI CSF samples using the validated and highly automated Roche Elecsys electrochemiluminescence immunoassays^30,31^ and the same reagent lot for each of these 3 biomarkers. Cerebrospinal fluid biomarker data were downloaded from the ADNI LONI site.^12^

### Statistical Analysis

Statistical analysis was conducted from November 1, 2017, to February 28, 2019. Logistic regression analysis was performed to explore the diagnostic group differences between AD diagnosis and each liver function marker separately. Age, sex, body mass index (BMI), and *APOE* ε4 status were used as covariates. We performed a linear regression analysis to access the association of liver function markers with composite scores for memory and executive functioning using age, sex, years of education, BMI, and *APOE* ε4 status as covariates. We also performed a linear regression analysis using age, sex, BMI, and *APOE* ε4 status as covariates.

#### ROI-Based Analysis of Structural MRI and PET Scans

Mean hippocampal volume was used as an MRI-related phenotype. For FDG-PET, a mean SUVR value was extracted from a global cortical ROI representing regions where patients with AD show decreased glucose metabolism relative to CN participants from the full ADNI-1 cohort, normalized to pons.^29^ For [^18^F]florbetapir PET, a mean SUVR value was extracted using MarsBaR from a global cortical region generated from an independent comparison of ADNI-1 [^11^C] Pittsburgh Compound B SUVR scans (regions where AD > CN). We performed a linear regression analysis using age, sex, BMI, and *APOE* ε4 status as covariates to evaluate the association of liver function markers with AD-related endophenotypes from MRI and PET scans. For hippocampal volume, years of education, intracranial volume, and magnetic field strength were added as additional covariates.^32^

#### Whole-Brain Imaging Analysis

The SurfStat software package^33^ was used to perform a multivariable analysis of cortical thickness to examine the association of liver function markers with brain structural changes on a vertex-by-vertex basis using a general linear model approach.^28^ General linear models were developed using age, sex, years of education, intracranial volume, BMI, *APOE* ε4 status, and magnetic field strength as covariates. The processed FDG-PET and [^18^F]florbetapir PET images were used to perform a voxelwise statistical analysis of the association of liver function markers with brain glucose metabolism and amyloid-β accumulation across the whole brain using SPM8.^34^ We performed a multivariable regression analysis using age, sex, BMI, and *APOE* ε4 status as covariates. In the whole-brain surface-based analysis, the adjustment for multiple comparisons was performed using the random field theory correction method with *P* < .05 adjusted as the level for significance.^35,36,37^ In the voxelwise whole-brain analysis, the significant statistical parameters were selected to correspond to a threshold of *P* < .05 (false discovery rate [FDR]–corrected).^38^

#### Multiple Testing Correction

Results of the analysis of liver function markers with AD diagnosis groups, cognitive composite measures, and A/T/N biomarkers for AD separately were corrected for multiple testing using the FDR with the Benjamini-Hochberg procedure (p.adjust command in R [R Project for Statistical Computing]).

## Results

### Study Sample

Our analyses included 1581 ADNI participants (407 CN, 20 with SMC, 298 with early MCI, 544 with late MCI, and 312 with AD). Demographic information as well as mean and SD of liver function markers stratified by clinical diagnosis are presented in eTable 2 in the Supplement.

### Diagnostic Group Difference of Liver Function Markers With AD Diagnosis

Levels of ALT were significantly decreased in AD compared with CN (odds ratio, 0.133; 95% CI, 0.042-0.422; *P* = .004) (Table 1), while AST to ALT ratio values were significantly increased in AD (odds ratio, 7.932; 95% CI, 1.673-37.617; *P* = .03). There was a trend to suggest that ALT levels were increased and AST to ALT ratio values were decreased in MCI compared with CN, but these became nonsignificant after adjustment for multiple comparisons (eTable 3 in the Supplement).

**Table 1.** Results of Association of Liver Function Biomarkers With Alzheimer Disease Diagnosis^a^

Liver Function Marker	Odds Ratio (95% CI)	Corrected *P* Value
Albumin, g/dL	5.789 (0.040-843.993)	.49
Alkaline phosphatase, U/L	3.620 (0.844-15.529)	.12
ALT, U/L	0.133 (0.042-0.422)	.004
AST, U/L	0.229 (0.045-1.175)	.12
AST to ALT ratio	7.932 (1.673-37.617)	.03
Total bilirubin, mg/dL	1.405 (0.585-3.377)	.49

Abbreviations: ALT, alanine aminotransferase; AST, aspartate aminotransferase.

^a^ Cognitively normal vs Alzheimer disease. Analyses were adjusted for age, sex, body mass index, and *APOE* ε4 status.

### Cognition

After adjusting for multiple comparison correction using FDR, we identified significant associations of liver function markers with cognition (Table 2). Higher levels of alkaline phosphatase and AST to ALT ratio were associated with lower memory scores (alkaline phosphatase: β [SE], –0.416 [0.162]; *P* = .02; AST to ALT ratio: β [SE], –0.465 [0.180]; *P* = .02) and executive functioning scores (alkaline phosphatase: β [SE], –0.595 [0.193]; *P* = .006; AST to ALT ratio: β [SE], –0.679 [0.215]; *P* = .006). Higher ALT levels were associated with higher memory scores (β [SE], 0.397 [0.128]; *P* = .006) and executive functioning scores (β [SE], 0.637 [0.152]; *P* < .001), whereas higher AST levels were associated with higher executive functioning scores (β [SE], 0.607 [0.215]; *P* = .01).

**Table 2.** Results of Association of Liver Function Biomarkers With Composite Cognitive Performance Measures^a^

Liver Function Marker	Memory Composite Score		Executive Function Composite Score	
	β (SE)	Corrected *P* Value	β (SE)	Corrected *P* Value
Albumin, g/dL	−0.872 (0.576)	.17	−0.203 (0.689)	.77
Alkaline phosphatase, U/L	−0.416 (0.162)	.02	−0.595 (0.193)	.006
ALT, U/L	0.397 (0.128)	.006	0.637 (0.152)	<.001
AST, U/L	0.339 (0.180)	.09	0.607 (0.215)	.01
AST to ALT ratio	−0.465 (0.180)	.02	−0.679 (0.215)	.006
Total bilirubin, mg/dL	−0.068 (0.103)	.61	−0.066 (0.123)	.65

Abbreviations: ALT, alanine aminotransferase; AST, aspartate aminotransferase.

^a^ Analyses were adjusted for age, sex, educational level, body mass index, and *APOE* ε4 status.

### Biomarkers of Amyloid-β

**Figure 1.**
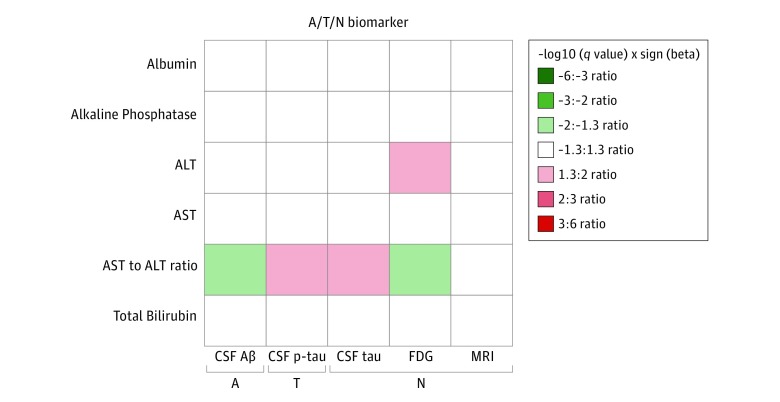
Results of Association of Liver Function Biomarkers With Amyloid, Tau, and Neurodegeneration (A/T/N) Biomarkers for Alzheimer Disease Heat map of q-values of the association between liver function markers and the A/T/N biomarkers for Alzheimer disease. *P* values estimated from linear regression analyses were corrected for multiple testing using false discovery rate (*q* value). White indicates *q* > 0.05, red indicates significant positive association, and green indicates significant negative association. Aβ indicates amyloid-β; ALT, alanine aminotransferase; AST, aspartate aminotransferase; CSF, cerebrospinal fluid; FDG, fludeoxyglucose positron emission tomography; MRI, magnetic resonance imaging; and p-tau, phosphorylated tau.

We used CSF amyloid-β 1-42 levels and a global cortical amyloid deposition measured from amyloid PET scans as biomarkers of amyloid-β. The regression coefficient of the AST to ALT ratio showed a negative association with CSF amyloid-β 1-42 levels (β [SE], –0.170 [0.061]; *P* = .04), indicating that higher AST to ALT ratio values were associated with CSF amyloid-β 1-42 positivity (Figure 1). However, there was no significant correlation between liver function markers and global cortical amyloid deposition.

**Figure 2.**
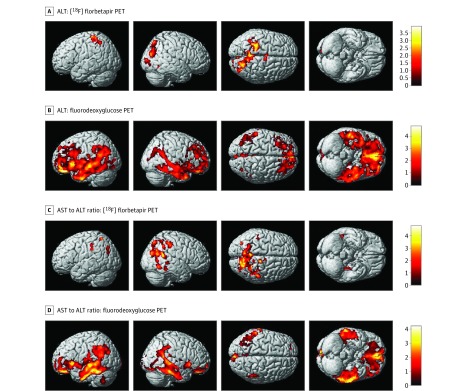
Detailed Whole-Brain Voxel-Based Imaging Analysis for Alanine Aminotransferase (ALT) and Aspartate Aminotransferase (AST) to ALT Ratio Levels Using Positron Emission Tomography (PET) Scans Whole-brain multivariable analysis was performed to visualize the topography of the association of ALT levels and AST to ALT ratio values with amyloid-β load and glucose metabolism on a voxelwise level (false discovery rate–corrected *P* < .05). A, Higher ALT levels were significantly associated with reduced amyloid-β deposition in the bilateral parietal lobes. B, Increased ALT levels were significantly associated with increased glucose metabolism in a widespread manner, especially in the bilateral frontal, parietal, and temporal lobes. C, Increased AST to ALT ratio values were significantly associated with increased amyloid-β deposition in the bilateral parietal lobes and the right temporal lobe. D, Increased AST to ALT ratio values were significantly associated with reduced brain glucose metabolism in the bilateral frontal, parietal, and temporal lobes.

In the whole-brain analysis using multivariable regression models to determine the association of liver function markers with amyloid-β load measured from amyloid PET scans on a voxelwise level, we identified significant associations for 2 liver function markers. Higher ALT levels were significantly associated with reduced amyloid-β deposition in the bilateral parietal lobes (Figure 2A). Increased AST to ALT ratio values were significantly associated with increased amyloid-β deposition in the bilateral parietal lobes and right temporal lobe (Figure 2C).

### Biomarkers of Fibrillary Tau

We used CSF p-tau levels as a biomarker of fibrillary tau. We investigated the association of liver function markers with CSF p-tau, adjusting for *APOE* ε4 status as a covariate. Higher AST to ALT ratio values were associated with higher CSF p-tau values (β [SE], 0.175 [0.055]; *P* = .02) (Figure 1).

### Biomarkers of Neurodegeneration or Neuronal Injury

We used structural atrophy measured from MRI scans, brain glucose metabolism from FDG-PET scans, and CSF t-tau levels as biomarkers of neurodegeneration or neuronal injury.

#### Brain Glucose Metabolism

We performed an ROI-based association analysis of liver function markers with a global cortical glucose metabolism value measured from FDG-PET scans across 1167 ADNI participants with both FDG-PET scans and measurement of liver function markers. The association analysis including *APOE* ε4 status as a covariate identified 2 markers as significantly associated with brain glucose metabolism after controlling for multiple testing using FDR (Figure 1). For ALT, higher levels were associated with increased glucose metabolism (β [SE], 0.096 [0.030]; *P* = .02), while for the AST to ALT ratio, higher ratio values were associated with reduced glucose metabolism (β [SE], –0.123 [0.042]; *P* = .03).

In the detailed whole-brain analysis to determine the association of liver function markers with brain glucose metabolism on a voxelwise level, increased ALT levels were associated with increased glucose metabolism in a widespread pattern, especially in the bilateral frontal, parietal, and temporal lobes (Figure 2B). However, higher AST to ALT ratio values were significantly associated with reduced glucose metabolism in the bilateral frontal, parietal, and temporal lobes (Figure 2D).

#### Structural MRI (Atrophy)

**Figure 3.**
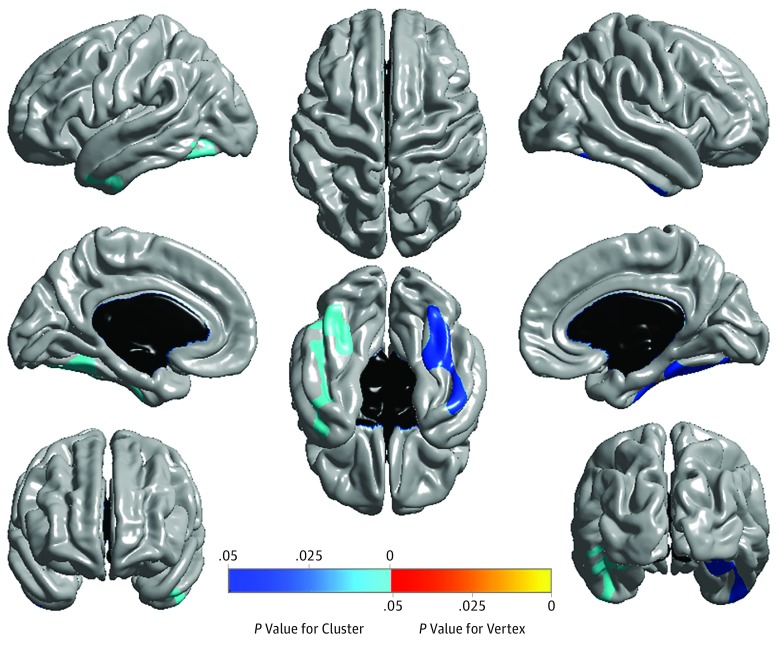
Detailed Whole-Brain Surface-Based Imaging Analysis for Alanine Aminotransferase (ALT) Levels Using Magnetic Resonance Imaging (MRI) Scans A whole-brain multivariable analysis of cortical thickness across the brain surface was performed to visualize the topography of the association of ALT levels with brain structure. Statistical maps were thresholded using a random field theory for a multiple testing adjustment to a corrected significance level of *P* < .05. The *P* value for clusters indicates significant corrected *P* values with the lightest blue color. Higher ALT levels were significantly associated with greater cortical thickness, especially in bilateral temporal lobes.

In the investigation of the association of liver function markers with mean hippocampal volume with *APOE* ε4 status as a covariate, we did not identify any significant association with hippocampal volume after controlling for multiple testing using FDR (Figure 1). Following the detailed whole-brain surface-based analysis of liver function markers using multivariable regression models to assess associations with cortical thickness, higher ALT levels were significantly associated with larger cortical thickness in the bilateral temporal lobes (Figure 3), which showed consistent patterns in the associations of brain glucose metabolism.

#### CSF t-Tau

Higher AST to ALT ratio values were associated with higher CSF t-tau levels (β [SE], 0.160 [0.049]; *P* = .02) (Figure 1), which showed consistent patterns in the associations of CSF amyloid-β 1-42 or p-tau levels and brain glucose metabolism.

## Discussion

We investigated the association between serum-based liver function markers and AD diagnosis, cognition, and AD pathophysiological characteristics based on the A/T/N framework for AD biomarkers in the ADNI cohort.^39^ Our findings suggest that the decreased levels of ALT and elevated AST to ALT ratio that were observed in patients with AD were associated with poor cognition and reduced brain glucose metabolism. We also found that an increased AST to ALT ratio was associated with lower CSF amyloid-β 1-42 levels, greater amyloid-β deposition, and higher CSF p-tau and t-tau levels. Furthermore, we observed that decreased levels of ALT were associated with greater amyloid-β deposition and structural atrophy.

Decreased levels of ALT and increased AST to ALT ratio values were observed in patients with AD and were associated with lower scores on measures of memory and executive function. Our findings are comparable with those of an earlier study that reported increased AST to ALT ratio values and lower levels of ALT in patients with AD compared with controls, although in that study, the association between AD and ALT levels did not reach statistical significance.^40^ Altered liver enzymes lead to disturbances in liver-associated metabolites including branched-chain amino acids, ether-phosphatidylcholines, and lipids,^41^ which we and others show are altered in AD^1,42,43,44^ and may play a role in disease pathophysiologic characteristics.^45^ Disturbed energy metabolism is one of the processes that may explain the observed lower levels of ALT and increased enzyme ratio in individuals with AD and impaired cognition.^3,5^ This finding is concordant with our observation that increased AST to ALT ratio values and lower levels of ALT showed a consistent significant association with reduced brain glucose metabolism, particularly in the orbitofrontal cortex and temporal lobes, areas of the brain implicated in memory and executive function. Brain glucose hypometabolism is an early feature of AD and cognitive impairment during the prodromal stage.^46,47^ Moreover, ALT and AST are key enzymes in gluconeogenesis in the liver and production of neurotransmitters required in maintaining synapses.^48^ Alanine aminotransferase catalyzes a reversible transamination reaction between alanine and α-ketoglutarate to form pyruvate and glutamate, while AST catalyzes a reversible reaction between aspartate and α-ketoglutarate to form oxaloacetate and glutamate.^49^ Although exact mechanisms remain unclear, 2 possible mechanisms may explain altered levels of enzymes in AD. First, reduced ALT levels lead to reduced pyruvate, which is required for glucose production via gluconeogenesis in the liver and glucose is distributed in various body tissues as an energy source,^50^ thus disturbing energy homeostasis. Second, altered levels of ALT and AST may affect levels of glutamate, an excitatory neurotransmitter of the central nervous system involved in synaptic transmission, which also plays an important role in memory.^51^

In the case of low glucose metabolism in the brain, as observed in our current study, less α-ketoglutarate is available via the tricarboxylic acid cycle that favors glutamate catabolism vs glutamate synthesis in reversible reaction (catalyzed by AST and ALT).^52^ Glutamate acts as a neurotransmitter in approximately two-thirds of the synapses in neocortical and hippocampal pyramidal neurons and thus is involved in memory and cognition via long-term potentiation.^53^ In a sample of healthy adults, plasma ALT and AST levels were significantly positively correlated with plasma glutamate levels,^5,54^ which indicates that lower levels of ALT will decrease glutamate levels in plasma. Based on evidence from earlier studies that peripheral blood levels of glutamate are positively correlated with levels of glutamate in the CSF^55^ and studies that reported lower levels of glutamate in patients with AD compared with controls in both blood^56^ and brain tissues,^36,57,58,59^ we can infer that lower levels of ALT or AST may affect glutamate levels in AD. In older adults, lower serum ALT levels are associated with mortality^60,61^ and are thought to be a biomarker for increased frailty, sarcopenia, and/or reduced levels of pyridoxine (vitamin B_6_).^62^ Pyridoxine phosphate is a coenzyme for the synthesis of amino acids, neurotransmitters (eg, serotonin and norepinephrine), and sphingolipids. Alanine aminotransferase decreases with age^63^ and may be a sign of hepatic aging. Glutamate levels also decrease with increasing age.^64^ Together with the fact that age is the strongest risk factor for AD,^65^ decreasing levels of ALT with age may also indicate a possible biological link between aging and AD. Nevertheless, further research is needed to determine the exact cause of reducing ALT levels with age and the pathway through which it can influence neurologic disorders, including AD.

Increased AST to ALT ratios are observed in individuals with nonalcoholic fatty liver disease, which is the hepatic manifestation of metabolic syndrome.^66^ In the Framingham Heart Study, nonalcoholic fatty liver disease was associated with smaller total cerebral brain volume even after adjustment for multiple cardiovascular risk factors.^67^ Liver dysfunction is also associated with the development of disease including cardiovascular disease and insulin resistance through disruptions in glucose and lipid metabolism, key physiological functions of the liver.^68,69^ Thus, using the AST to ALT ratio as a marker for overall metabolic disturbance,^5^ our study provides evidence of an association between altered metabolic status and AD, cognition, and AD endophenotypes.

In addition to ALT levels and the AST to ALT ratio, elevated levels of alkaline phosphatase were significantly associated with poor cognition. This is in line with results from the Oxford Project to Investigate Memory and Aging, which reported increased alkaline phosphatase levels in individuals with AD and an inverse association with cognition.^70^ Alkaline phosphatase is an enzyme primarily expressed in the liver and kidneys as well as in endothelial cells in the brain.^71,72^ The neuronal form of alkaline phosphatase plays a role in developmental plasticity and activity-dependent cortical functions via contributing in γ-aminobutyric acid metabolism.^73,74,75,76^ Changes in plasma levels of alkaline phosphatase may occur as a result of central nervous system injury.^77^

### Limitations

This study has several limitations. The observational design of this ADNI cohort study limits our ability to make assumptions about causality. There is need to evaluate the association of liver enzymes with AD in prospective manner. Another limitation of our study is that we did not adjust for alcohol consumption, which was not available in ADNI. Alcohol consumption is associated with altered liver enzymes. Instead, we used a well-established surrogate marker of alcohol consumption, γ-glutamyltransferase. Elevations in γ-glutamyltransferase generally indicate long-term heavy drinking rather than episodic heavy drinking.^78^ Our key findings remained significant after adjustment for γ-glutamyltransferase and statin use (eTable 4, eTable 5, and eFigure in the Supplement). However, given the associations with liver function measures and A/T/N biomarkers for AD, it appears that liver function may play a role in the pathogenesis of AD, but limitations should be taken into account before further extrapolating our findings.

## Conclusions

This study’s results suggest that altered liver function markers are associated with AD diagnosis and impaired memory and executive function as well as amyloid-β, tau, and neurodegenerative biomarkers of AD pathophysiological characteristics. These results are, to our knowledge, the first to show an association of peripheral markers of liver functioning with central biomarkers associated with AD. Although our results suggest an important role of liver functioning in AD pathophysiological characteristics, the causal pathways remain unknown. The liver-brain biochemical axis of communication should be further evaluated in model systems and longitudinal studies to gain deeper knowledge of causal pathways.

## Notes

Supplement. **eTable 1.** Sensitivity Analysis for Alzheimer Disease Diagnosis (CN vs AD) Group Differences in Liver Function Biomarkers **eTable 2.** Demographic Information of ADNI Participants **eTable 3.** Diagnostic Group Differences in Liver Function Biomarkers **eTable 4.** Diagnostic Group Differences of Liver Function Biomarkers With Alzheimer Disease Diagnosis (CV vs AD) Adjusted for Gamma-Glutamyltransferase (GGT) and Statin Use **eTable 5.** Association of Liver Function Biomarkers With Cognition Adjusted for Gamma-Glutamyltransferase (GGT) and Statin Use **eFigure.** Liver Function Biomarkers and Their Association With A/T/N Biomarkers for Alzheimer Disease Adjusted for Gamma-Glutamyltransferase (GGT) and Statin Use
