# Supplementary material for: Association of Altered Liver Enzymes With Alzheimer Disease Diagnosis, Cognition, Neuroimaging Measures, and Cerebrospinal Fluid Biomarkers
Source: JAMA Netw Open. 2019 Jul 31;2(7):e197978. doi: 10.1001/jamanetworkopen.2019.7978 (PMC6669786; doi:10.1001/jamanetworkopen.2019.7978)
Supplement: Supplement. — eTable 1. Sensitivity Analysis for Alzheimer Disease Diagnosis (CN vs AD) Group Differences in Liver Function Biomarkers eTable 2. Demographic Information of ADNI Participants eTable 3. Diagnostic Group Differences in Liver Function Biomarkers eTable 4. Diagnostic Group Differences of Liver Function Biomarkers With Alzheimer Disease Diagnosis (CV vs AD) Adjusted for Gamma-Glutamyltransferase (GGT) and Statin Use eTable 5. Association of Liver Function Biomarkers With Cognition Adjusted for Gamma-Glutamyltransferase (GGT) and Statin Use eFigure. Liver Function Biomarkers and Their Association With A/T/N Biomarkers for Alzheimer Disease Adjusted for Gamma-Glutamyltransferase (GGT) and Statin Use [file jamanetwopen-2-e197978-s001.pdf]

Supplementary Online Content

Nho K, Kueider-Paisley A, Ahmad S, et al; Alzheimer's Disease Neuroimaging Initiative and the Alzheimer Disease Metabolomics Consortium. Association of altered liver enzymes with Alzheimer disease diagnosis, cognition, neuroimaging measures, and cerebrospinal fluid biomarkers. *JAMA Netw Open.* 2019;2(7):e197978.
doi:10.1001/jamanetworkopen.2019.7978

eTable 1. Sensitivity Analysis for Alzheimer Disease Diagnosis (CN vs AD) Group Differences in Liver Function Biomarkers
eTable 2. Demographic Information of ADNI Participants
eTable 3. Diagnostic Group Differences in Liver Function Biomarkers
eTable 4. Diagnostic Group Differences of Liver Function Biomarkers With Alzheimer Disease Diagnosis (CV vs AD) Adjusted for Gamma-Glutamyltransferase (GGT) and Statin Use
eTable 5. Association of Liver Function Biomarkers With Cognition Adjusted for Gamma-Glutamyltransferase (GGT) and Statin Use
eFigure. Liver Function Biomarkers and Their Association With A/T/N Biomarkers for Alzheimer Disease Adjusted for Gamma-Glutamyltransferase (GGT) and Statin Use

This supplementary material has been provided by the authors to give readers additional information about their work.

© 2019 Nho K et al. *JAMA Network Open.*

eTable 1. Sensitivity Analysis for Alzheimer Disease Diagnosis (CN vs AD) Group Differences in Liver Function Biomarkers

Liver function marker	excluding outliers			keeping outliers		
	Odds Ratio	95% CI	Corrected *P* Value	Odds Ratio	95% CI	Corrected *P* Value
**Albumin**	5.789	0.040,843.993	4.90×10^{-1}	5.789	0.040,843.993	4.90×10^{-1}
**Alkaline Phosphatase**	3.620	0.844,15.529	1.25×10^{-1}	3.620	0.844,15.529	1.25×10^{-1}
**ALT**	0.133	0.042,0.422	3.63×10^{-3}	0.120	0.039,0.374	2.55×10^{-3}
**AST**	0.229	0.045,1.175	1.25×10^{-1}	0.203	0.041,1.021	1.06×10^{-1}
**AST:ALT**	7.932	1.673,37.617	2.73×10^{-2}	9.815	2.156,44.670	9.41×10^{-3}
**Total Bilirubin**	1.405	0.585,3.377	4.90×10^{-1}	1.405	0.585,3.377	4.90×10^{-1}

Abbreviations: AST: Aspartate aminotransferase; ALT: alanine aminotransferase. Age, sex, body mass index, and *APOE* ε4 status included as covariates

© 2019 Nho K et al. *JAMA Network Open.*

eTable 2. Demographic Information of ADNI Participants

Variable	CN (N=407)	SMC (N=20)	EMCI (N=298)	LMCI (N=544)	AD (N=312)	*P* Value
**Age (years)**	74.5 (5.7)	71.6 (5.1)	70.9 (7.4)	73.5 (7.6)	74.6 (7.7)	1.94×10^{-11}
**Sex: (Male/Female)**	206/201	10/10	166/132	331/213	171/141	3.35×10^{-2}
**Education (years)**	16.3 (2.7)	16.8 (2.2)	16.0 (2.6)	15.9 (3.0)	15.1 (3.0)	1.59×10^{-6}
**BMI (Kg/M^2)**	27.4 (4.5)	29.3 (4.6)	28.4 (5.3)	26.9 (4.3)	26.1 (4.4)	6.94×10^{-9}
***APOE* ε4 status (0/1)**	294/113	15/5	169/129	249/295	98/214	$<2.20 \times 10^{-16}$
**Albumin (g/dL)**	4.09 (0.31)	3.94 (0.25)	4.10 (0.29)	4.14 (0.32)	4.11 (0.32)	1.29×10^{-2}
**Alkaline phosphatase (U/L)**	75.3 (20.7)	74.4 (14.1)	71.1 (17.9)	74.9 (22.4)	76.2 (19.2)	2.09×10^{-2}
**ALT (U/L)**	21.5 (8.4)	24.2 (6.6)	22.2 (9.3)	20.7 (8.0)	19.5 (7.0)	5.44×10^{-5}
**AST (U/L)**	24.3 (6.2)	25.2 (8.0)	24.7 (7.4)	24.1 (6.1)	23.7 (6.3)	4.66×10^{-1}
**AST:ALT**	1.22 (0.32)	1.06 (0.22)	1.18 (0.31)	1.25 (0.31)	1.29 (0.35)	1.22×10^{-5}
**Total bilirubin (mg/dL)**	0.52 (0.26)	0.44 (0.14)	0.54 (0.28)	0.53 (0.24)	0.56 (0.30)	3.02×10^{-1}
**Memory composite score**	0.94 (0.53)	0.99 (0.57)	0.53 (0.49)	-0.04 (0.57)	-0.77 (0.54)	$<2.20 \times 10^{-16}$
**Executive function composite score**	0.75 (0.71)	0.82 (0.64)	0.47 (0.76)	0.01 (0.80)	-0.88 (0.82)	<2.20x<1$0^{-16}$

Abbreviations: AD: Alzheimer's Disease; ALT: alanine aminotransferase; AST: Aspartate aminotransferase; BMI: Body Mass Index; CN: Cognitively Normal; EMCI: early mild cognitive impairment; LMCI: late MCI; SMC: subjective memory concerns

© 2019 Nho K et al. *JAMA Network Open.*

eTable 3. Diagnostic Group Differences in Liver Function Biomarkers

Liver function marker	CN vs EMCI		CN vs LMCI		CN vs AD	
	Odds Ratio	95% CI	Odds Ratio	95% CI	Odds Ratio	95% CI
**Albumin (g/dL)**	0.811	0.006,117.8	19.623	0.333,1156.4	5.789	0.040,844.0
**Alkaline Phosphatase (U/L)**	0.144	0.035,0.588	0.883	0.290,2.694	3.620	0.844,15.53
**ALT (U/L)**	0.561	0.196,1.604	0.291	0.112,0.755	0.133	0.042,0.422
**AST (U/L)**	0.742	0.170,3.230	0.393	0.102,1.506	0.229	0.045,1.175
**AST:ALT**	1.240	0.278,5.540	3.783	0.995,14.38	7.932	1.673,37.62
**Total Bilirubin (mg/dL)**	1.319	0.556,3.133	0.887	0.417,1.886	1.405	0.585,3.377

Abbreviations: AD: Alzheimer's Disease; ALT: alanine aminotransferase; AST: Aspartate aminotransferase; BMI: Body Mass Index; CN: Cognitively Normal; EMCI: early mild cognitive impairment; LMCI: late MCI. Age, sex, body mass index, and APOE ε4 status were included as covariates.

© 2019 Nho K et al. *JAMA Network Open.*

eTable 4. Diagnostic Group Differences of Liver Function Biomarkers With Alzheimer Disease Diagnosis (CV vs AD) Adjusted for Gamma-Glutamyltransferase (GGT) and Statin Use

**Liver function marker**	**Odds Ratio**	**95% CI**	**Corrected *P* Value**
**Albumin (g/dL)**	5.595	0.037, 840.4	5.01×10^{-1}
**Alkaline Phosphatase (U/L)**	4.296	0.982, 18.79	1.06×10^{-1}
**ALT (U/L)**	0.112	0.032, 0.386	3.16×10^{-3}
**AST (U/L)**	0.246	0.045, 1.348	1.59×10^{-1}
**AST:ALT**	8.580	1.713, 42.98	2.68×10^{-2}
**Total Bilirubin (mg/dL)**	1.461	0.603, 3.539	4.82×10^{-1}

Abbreviations: AST: Aspartate aminotransferase; ALT: alanine aminotransferase

© 2019 Nho K et al. *JAMA Network Open.*

eTable 5. Association of Liver Function Biomarkers With Cognition Adjusted for Gamma-Glutamyltransferase (GGT) and Statin Use

**Liver function markers**	**Memory**			**Executive function**		
	**β**	**SE**	**Corrected *P* Value**	**β**	**SE**	**Corrected *P* Value**
**Albumin (g/dL)**	-0.843	0.578	1.93×10^{-1}	-0.238	0.691	7.31×10^{-1}
**Alkaline Phosphatase (U/L)**	-0.427	0.163	1.51×10^{-2}	-0.607	0.194	5.52×10^{-3}
**ALT (U/L)**	0.476	0.139	3.62×10^{-3}	0.733	0.165	1.18×10^{-4}
**AST (U/L)**	0.366	0.187	7.62×10^{-2}	0.631	0.224	1.16×10^{-2}
**AST:ALT**	-0.511	0.188	1.33×10^{-2}	-0.709	0.224	5.52×10^{-3}
**Total Bilirubin (mg/dL)**	-0.066	0.103	6.22×10^{-1}	-0.070	0.123	6.22×10^{-1}

Abbreviations: AST: Aspartate aminotransferase; ALT: alanine aminotransferase; GGT: gamma-glutamyl transferase. Covariates included age, sex, education, body mass index, *APOE* ε4 status, statins use, and gamma-glutamyl transferase.

© 2019 Nho K et al. *JAMA Network Open.*

eFigure. Liver Function Biomarkers and Their Association With A/T/N Biomarkers for Alzheimer Disease Adjusted for Gamma-Glutamyltransferase (GGT) and Statin Use

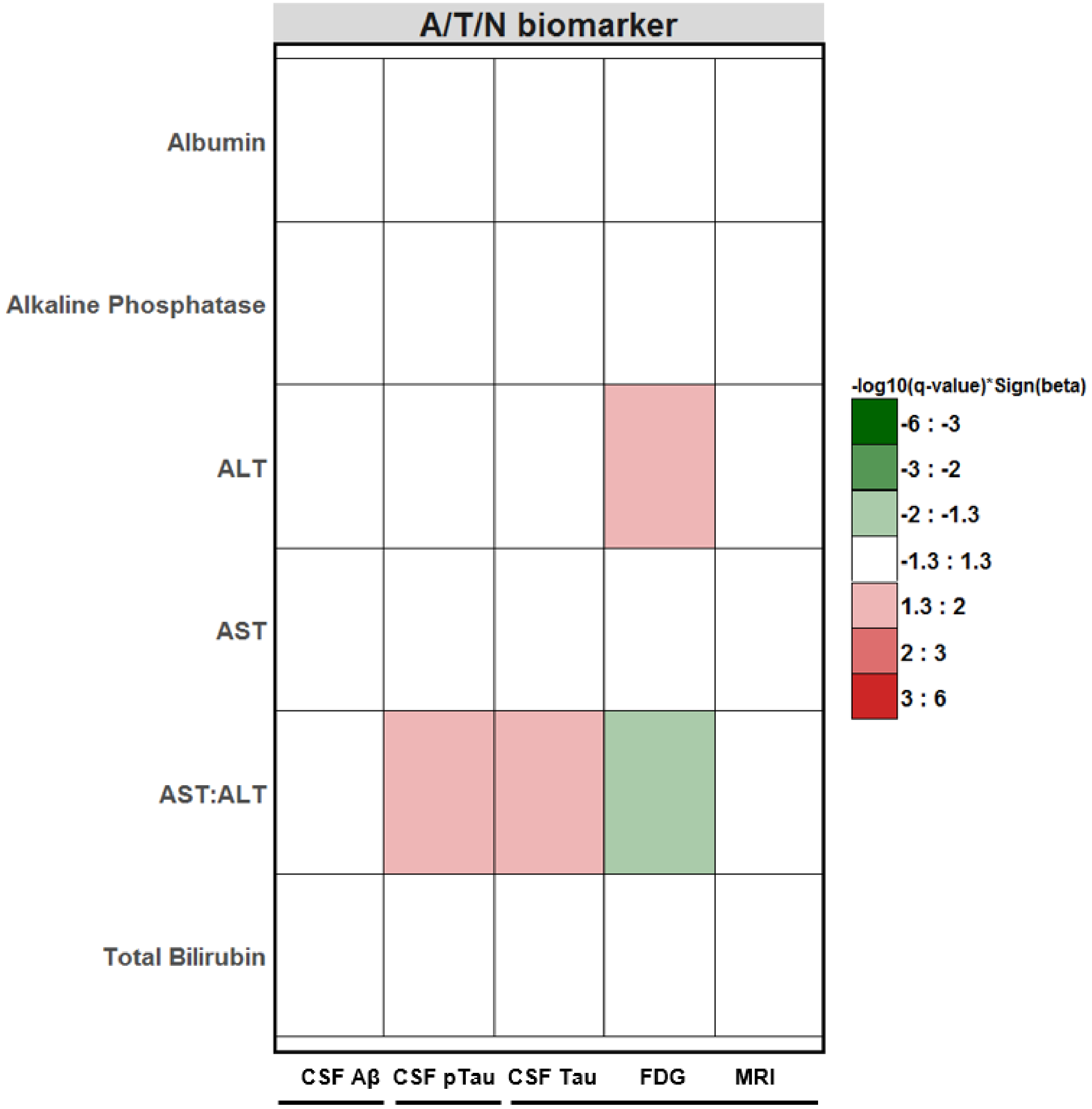

Supplementary Figure 1: Liver function biomarkers and their association with ATN biomarkers for Alzheimer's disease adjusted for statin use and gamma-glutamyltransferase. Heat map of q-values of association between liver function biomarkers and the "A/T/N" biomarkers for Alzheimer's disease. P-values estimated from linear regression analyses were corrected for multiple testing using FDR (q-value). Color code: white indicates q-value>0.05, red indicates significant positive associations, and green indicates significant negative associations.

© 2019 Nho K et al. *JAMA Network Open.*
